# University of Buffalo—Denial Department

**Published:** 1895-06

**Authors:** 


### COMMENCEMENTS.

### University of Buffalo—Dental Department.

The third annual commencement exercises were held in connection with the Departments of Medicine and Pharmacy at Music Hall, in the city of Buffalo, on Tuesday, April 30th, 1895. After thorough examination by the board of curators, including the State Dental Examining Board, the Chancellor upon their recommendation conferred the degree of D.D.S. upon the following members of the senior class:

Will B. Bartlett.
Charles Frederick Bunbury.
Gerald Griffin Burns.
Charles Alexander Bradshaw.
Fred. Ellsworth Cloud.
Thomas James Dorland.
Charles Edwin Flagg.
Frederic Jacob Gieser.
William George Gowland.
Perry Charles Hammersmith.
Frank Lewis Haynes.
Albert Heinrich.
Lott Myron Howder.
George Lintner Hussong.
Harry Bestow Huver.
Lloyd Starr Ingalls.
William Erastus Jenney.
Clint Wood La Salle.
Charles Thomas Lewis.
John James Madden.
Henry James McLellan.
Alfred Edward Mimmack.
Bertram Amos Moyer.
Thomas Clarence Phillips.
Miss Annette Rankin.
Albert Boniface Rieger.
Frank Mills Rowland.
John Wesley Steele.
Harry George Tripp.
Burdette Washington Whipple.

The whole number of matriculates for the session was 160.

